# Comparison of efficacy and safety of complementary and alternative therapies for primary trigeminal neuralgia: A protocol for a Bayesian network meta analysis

**DOI:** 10.1097/MD.0000000000024212

**Published:** 2021-01-15

**Authors:** Tianqi Zhang, Tiefeng Zhang, Chuancheng Li, Xixi Zhai, Qing Huo

**Affiliations:** a First College of Clinical Medicine, Shandong University of Traditional Chinese Medicine; b Second Affiliated Hospital of Shandong University of traditional Chinese Medicine; c Affiliated Hospital of Shandong University of Traditional Chinese Medicine, Jinan, Shandong Province, China.

**Keywords:** Bayesian, complementary and alternative therapies, network meta-analysis, primary trigeminal neuralgia, protocol

## Abstract

**Background:**

Primary trigeminal neuralgia (PTN) is a type of peripheral neuralgia that seriously affects people's lives. In recent years, complementary therapies and alternative therapies have played a significant role in treating PTN. However, there is a lack of comparison among all the complementary and alternative therapies at present. Thus, the aim of this study is to discuss the efficacy and safety of diverse complementary and alternative therapies by Bayesian network meta-analysis (NMA).

**Methods:**

We will retrieve the Chinese and English databases to gather related randomized controlled trials (RCTs) of complementary and alternative therapies for treating PTN. The deadline is November 2020. Two independent researchers will be in charge of screening qualified literature, extracting data, and independently evaluating bias risks involved in the research. Pairwise meta-analysis and Bayesian network meta-analysis will be performed to assess all evidence. Then, we will use STATA16.0 as well as WinBUGS1.4.3 software for data analysis. Besides, the quality of NMA evidence will be classified by grading of recommendations assessment development and evaluation (GRADE).

**Results:**

This study will compare and rank the efficacy and safety of different complementary and alternative therapies in treating primary trigeminal neuralgia.

**Conclusion:**

Complementary and alternative therapies play an essential role in treating primary trigeminal neuralgia. We expect our study will furnish meaningful evidence support for clinicians and patients.

**Protocol registration number:**

INPLASY2020120026.

**Ethical approval:**

Since the study is based on published or registered RCTs, ethical approval and patient informed consent are abandoned.

## 1 Introduction

Primary trigeminal neuralgia (PTN) is a kind of chronic peripheral neuropathic pain, characterized by transient and paroxysmal, electric-shock, knife-cutting, tearing-like severe pain, which seriously affects the quality of life and social activities of patients.^[1,2]^ Trigeminal neuralgia is uncommon, affecting 4 to 13 people per 100,000.^[3]^ The pathogenesis is still not clear, the influence of trigeminal neuralgia on women (60%) is more than that of men (40%). The average age of onset was reported to be 53 to 57 years.^[4]^ Moreover, an epidemiological study showed that anxiety and depression of PTN patients increased, which highlighted the impact on mental health.^[5]^

Generally speaking, drug therapy is the primary choice for PTN. The anticonvulsant agent carbamazepine and oxcarbazepine constitute the first-line medical treatment. However, clinical improvement is usually offset by side effects, including dizziness, ataxia, diplopia, and elevated aminotransferase levels, which may lead to treatment withdrawal in 23% of patients.^[2]^ Surgical treatment of PTN patients may be suitable for those who have failed to treat with at least 3 drugs, have intolerable side effects or symptoms that can’t be alleviated. At present, the main surgical treatment is microvascular decompression, radiofrequency thermocoagulation and radiotherapy, and so on. Microvascular decompression (MVD) is currently the safest and most effective surgical method. It is effective in 90% of patients in the short term, but the long-term effective rate is reduced to 50% to 70%, and the average annual recurrence rate is 3.5%.^[6]^ Complications such as hearing loss, cerebrospinal fluid leakage, temporary facial paralysis may occur after operation. Radiofrequency thermocoagulation (RFT) may lead to facial hypoesthesia, facial numbness, keratitis, and corneal reflex disappearance. Gamma knife stereotactic radiotherapy (GKSR) has the disadvantages of high recurrence rate and delayed pain control. Besides, the surgical treatment of PTN is difficult, dangerous, and destructive, and some complications of sensory and motor nerve injury will inevitably occur.

In short, PTN is mainly treated by western medicine, MVD, RFT, GKSR, and local blocking therapy. These therapies lack specific treatment, and also have adverse effects such as hearing loss, dizziness, sensory and motor nerve damage. Therefore, it is a treatment trend to seek green therapy with little trauma, better curative effects, and fewer side effects. Nowadays complementary and alternative therapies have been widely used with significant clinical effects, that usually include Chinese herbal medicine, acupuncture, moxibustion, massage, acupoint injection, psychotherapy, and so on. It is pointed out that acupuncture and traditional Chinese medicine have obvious advantages in treating PTN disease, which can not only reduce the pain degree and side effects of western medicine, but also improve the clinical curative effect, thus improving the quality of life of patients. TCM contains multiple components, which can target different pain pathological mechanisms. TCM can significantly improve the curative effect and help predict the risks of pain through various components and targets.^[7,8]^ Latest research indicates that acupuncture, a traditional Chinese practice, is much less stressful, safer, and cheaper than medication or surgery.^[9,10]^

Despite the effectiveness of randomized controlled trials (RCTs) and systematic reviews, it is hard for numerous doctors and patients to opt the most appropriate method. Because the traditional meta-analysis usually compares 2 kinds of interventions, how to select the most effective and safest methods has become a critical clinical problem in the face of various interventions. Network meta-analysis (NMA) can compare various interventions and then choose the best one. Therefore, this study is based on the existing RCTs to study the efficacy and safety of complementary and alternative therapies for PTN. We hope to provide clinical doctors and patients with scientific and rigorous evidence support.

## 2 Methods

This study will strictly follow the Preferred Reporting Items for Systematic Review and Meta-Analysis Protocols (PRISMA-P) to perform this study.^[11]^

### 2.1 Study Registration

The network meta-analysis has been registered on the international platform of registration system evaluation and meta-analysis agreement. The registration number is: INPLASY 2020120026 (URL = https://inplasy.com/inplasy-2020-12-0026/).

### 2.2 Inclusion criteria

#### 2.2.1 Type of study

This study will include RCTs and systematic review/meta-analysis of supplementary and alternative treatments in PTN treatment. Case reports, reviews, animal experiments, non-RCT, or semi-RCT trials, will not be included in this study. The language will be restricted in Chinese or English.

#### 2.2.2 Participants

(1) Patients diagnosed as primary trigeminal neuralgia;
(2) No restrictions on sex, age, race, nationality, etc.

#### 2.2.3 Interventions

In the treatment group, PTN must be treated with complementary therapy and alternative therapies on the basis of conventional western medicine, or be used alone. Complementary therapy and alternative therapies include Chinese herbal medicine, acupuncture, moxibustion, massage, acupoint injection, psychotherapy, etc. The control group should be treated with western medicine or other methods based on western medicine. Other methods include microvascular decompression, radiofrequency thermocoagulation, radiotherapy, local blocking therapy, and so on.

#### 2.2.4 Outcomes

The primary outcomes include the total efficiency rate and the visual analogue scale (VAS). VAS is a useful and reliable tool to determine the severity of pain, that ranges from 0 (no pain) to 10 (unbearable pain). The secondary outcomes include pain numerical rating scale (NRS), analgesic dose, recurrence rate, pain duration, and the incidence of toxic and side effects. Total efficiency rate will be calculated according to the total number of random patients.

### 2.3 Exclusion criteria

(1) Repeated publications;
(2) Case reports, reviews, animal experiments, non-RCT, semi-RCT trials, etc.
(3) Complete data cannot be obtained or the data are wrong.
(4) Secondary trigeminal neuralgia or other diseases.
(5) PTN combined with other pain-causing diseases in the literature.

### 2.4 Databases and search strategy

We will search all related RCTs according to the complementary and alternative therapies for PTN from inception to November 2020 in the following databases: PubMed, Cochrane Library, Cochrane Central Register of Controlled Trials, Web of Science, EMBASE, Chinese Biomedical Literature Database (SinoMed), CNKI, VIP Database, Wanfang Database. Meanwhile, we will follow up the references included in the systematic review/meta-analysis. The search strategy will be constructed by MeSH and keywords. The retrieval scheme of the PubMed database is shown in Table 1.

**Table 1:** Search strategy for PubMed.

No.	Search item
#1	Primary trigeminal neuralgia [MeSH Terms]
#2	trigeminal neuralgia [Title/Abstract] OR trigeminal [Title/Abstract] OR primary [Title/Abstract] OR neuralgia [Title/Abstract]
#3	#1 OR #2
#4	Complementary Therapies [MeSH Terms]
#5	Therapies, Complementary [Title/Abstract] OR Therapy, Complementary [Title/Abstract] OR Complementary Medicine [Title/Abstract] OR Medicine, Complementary [Title/Abstract] OR Alternative Medicine [Title/Abstract] OR Medicine, Alternative [Title/Abstract] OR Alternative Therapies [Title/ Abstract] OR Therapies, Alternative [Title/Abstract] OR Therapy, Alternative [Title/Abstract] OR Herb Therapy [Title/Abstract]
#6	#4 OR #5
#7	Chinese herbal medicines [Title/Abstract] OR Acupuncture [Title/Abstract] OR Moxibustion [Title/Abstract] OR Massage [Title/Abstract] OR Acupoint injection [Title/Abstract] OR psychotherapy [Title/Abstract]
#8	#6 OR #7
#9	Randomized Controlled Trial [Publication Type] OR Controlled Clinical Trial [Publication Type] OR Randomized [Title/Abstract] OR randomly [Title/Abstract] OR random allocation [Title/Abstract]
#10	#3 AND #8 AND #9

### 2.5 Data extraction

According to the above strategy, we will retrieve all related papers from the database and then import the research into Endnote X9. Then, 2 reviewers (TqZ and TfZ) will independently screen and extract data. All titles and abstracts of documents will be searched and irrelevant documents will be ruled out. For all abstracts deemed eligible for inclusion during the first level of review, full-text articles will be retrieved and reviewed. Full-text screening will be conducted by 2 independent investigators (TqZ and TfZ). We will resolve disagreements through discussion, or, if necessary, we will consult the third reviewer (CL). If the required information is not complete, we are going to contact the corresponding author. The data of literature will be extracted as following:

1. General information of the study, including title, first author, journal, publication date, registration number to trial registries, data extraction, country.
2. Basic information of participants, including sex, age, sociodemographic characteristics, sample size, source of research subjects, inclusion criteria, exclusion criteria, course of treatment, people lost to follow-up or dropped out.
3. Interventions measures, including therapeutic method, frequency, dosage, and the duration of treatment.
4. Outcomes, including primary outcomes (total efficiency rate and VAS), secondary outcomes (NRS, analgesic dose, recurrence rate, pain duration, incidence of toxic, and side effects).

### 2.6 Risk of bias assessment

The quality will be assessed using the Cochrane Collaboration's Risk of Bias Tool by 2 researchers independently.^[12]^ The main contents comprise 7 items and each item will be divided into 3 levels: “high,” “unclear,” and “low.”

### 2.7 Assessment of heterogeneity

There are differences inevitably owing to the variety of our study designs, similar studies from different countries or regions. We will apply the chi-square test to estimate heterogeneity. If the *I*^2^ of the whole network is <50%, it indicates that the heterogeneity is slight, then the fixed effect model will be used. If the *I*^2^ is >50%, the heterogeneity is distinct, we ought to make a comprehensive and systematic analysis of the causes. While ruling out all the heterogeneous factors, we will adopt a random-effects model.

### 2.8 Subgroup analysis and sensitivity analysis

Assuming heterogeneity exists in the research, we will deal with it in subgroup analysis according to various sources of heterogeneity. Besides, for different design schemes, we will make subgroup analysis in the light of design scheme, country, publication year, age, onset time, and duration.

In addition, we will analyze the sensitivity of all outcome indicators by the exclusion method to. If the heterogeneity changes after excluding an article, then this article is the cause of the heterogeneity. It can be discussed in terms of sample size, experimental design, result indicators, evaluation criteria, etc. On the contrary, if the heterogeneity still the same, then the result is stable and reliable.

### 2.9 Statistical analysis

#### 2.9.1 Pairwise meta-analysis

We will carry out STATA16.0 software for pairwise meta-analysis. Bivariate and continuous variables are represented by odds ratio (OR) and mean difference (MD) respectively. We will analyze 95% confidence interval (CI) for each effect indicator and calculate *I*^2^ for assessing the heterogeneity among studies.

#### 2.9.2 Network meta-analysis

We will adopt STATA 16.0 to draw a network diagram to compare the intervention measures of each outcome indicator. In addition, we are going to utilize WinBUGS1.4.3 software to analyze the data, using the Bayesian Markov Chain Monte Carlo (MCMC) random effect model.^[13]^ Bayesian NMA can settle statistical processing in sophisticated evidence networks, so it is much more flexible and efficient. Meanwhile, the posterior probabilities obtained can be used to rank all intervention from good to bad. When working WinBUGS1.4.3 program, for every MCMC, the number of iterations will be set to 50,000, of which the first annealing is set to 20,000 times to remove the influence of the initial value, as well as the final 30,000 times are sampled. We will assess convergence of the iteration by the Brooks-Gelman Rubin method. If the potential scale reduction factor (PSRF) tends to 1, it means that the convergence of the model is more reliable.^[14,15]^ What's more, we will modulate the iteration times and annealing times in terms of the actual situation, and calculate the corresponding effective value of 95% CI. Moreover, the intervention measures will be ranked by using the surface under the cumulative ranking curve (SUCRA) values.^[16]^ The SUCRA values range from 0 to 1. The closer to 1, the more likely the intervention will be the best one.

#### 2.9.3 Assessment of inconsistency

If NMA is closed loop, inconsistency will be estimated. Hence, we will calculate the difference between direct and indirect comparative evidence by the node splitting method. Then we will adopt the *P* value to conform whether there is inconsistency or not.^[17,18]^

### 2.10 Evaluation of publication bias and evidence quality

For articles with >10 researches, a comparison-correction funnel chart will be established for the outcome indicators. If the funnel plot is symmetric, there is no significant publication bias. If it is asymmetric, there is a publication bias, it indicates that there may be publication bias and then we will analyze the reasons for that. We will assess the quality of evidence by GRADE, which includes risk of bias, indirectness, inconsistency, imprecision, and publication bias.^[19]^

## 3 Discussion

PTN is one of the most common causes of facial pain, which means short-lasting episodes of unilateral electric shock-like pain with abrupt onset and termination.^[20,21]^ Drug therapy is the first choice for treating PTN. At present, carbamazepine and oxcarbazepine are the first-line drugs.^[22]^ After long-term medication, the curative effect will decline, and adverse reactions such as dizziness and headache will occur. When the effect of drug treatment is unsatisfactory, surgical treatment can be selected, such as microvascular decompression, radiofrequency thermocoagulation, and radiation therapy.^[23]^ However, there are some patient intolerant to the operation or have adverse effects such as hearing loss, facial nerve palsy, cerebrospinal fluid leakage, and so on.

Hence, in recent years, people have paid more and more attention to the supplementary therapies and replacement therapies for PTN.^[24]^ Complementary and alternative therapies commonly used include Chinese herbal medicine, acupuncture, massage, acupoint injection, psychotherapy, etc. Studies have found that these therapies can play a critical role in improving the symptoms of PTN, reducing the degree of pain and recurrence rate, and having fewer side effects.^[24–26]^ For example, TCM contains a variety of ingredients, which can effectively target different pathological mechanisms involved in pain and have significant analgesic effects.^[23,27,28]^ Recently, acupuncture has also been recognized by more and more people, and the effectiveness and safety of it have been increasingly verified.^[29,30]^

For clinicians, how to choose safer and more effective measures or their combination has become a key point to be solved urgently. As we all know, traditional meta-analysis primarily concentrates on the pairwise comparison of intervention measures, and can’t make multiple comparative analysis among various intervention measures. The network meta-analysis is based on indirect comparative meta-analysis or multiple intervention, which has obvious advantages. Our study is the first network meta-analysis on the intervention of complementary and alternative therapies for PTN. The aim of the research is to supply evidence for the effectiveness and safety of these therapies in treating PTN. In this study, we will perform a comprehensive and systematic literature search, and conduct a network meta-analysis using Bayesian models, rank interventions through SURCA, and adopt GRADE to assess the quality of the evidence.

Although NMA has many advantages, there are still some limitations in our research. For example, our research is in view of literature not the original data, so there will be some deviations. Meanwhile, the quality of literature research is varied. Thus, we hope to include more large-scale, high-quality RCTs to improve the level of the evidence-based medicine continuously. We expect to assist clinicians and patients to choose the optimum complementary and alternative treatments for PTN, and provide better support for clinical practice.

## Author contributions

**Conceptualization:** Tianqi Zhang, Tiefeng Zhang, Qing Huo.

**Formal analysis:** Tianqi Zhang, Tiefeng Zhang.

**Methodology:** Chuancheng Li, Xixi Zhai.

**Project administration:** Qing Huo.

**Software:** Xixi Zhai, Tianqi Zhang.

**Writing – original draft:** Tianqi Zhang, Tiefeng Zhang.

**Writing – review & editing:** Tianqi Zhang, Qing Huo.

## Abbreviations

Abbreviations: CI = confidence interval, GRADE = grading of recommendations assessment development and evaluation, MeSH = medical subject headings, NMA = network meta-analysis, OR = odds ratio, PTN = primary trigeminal neuralgia, RCT = randomized controlled trials.
